# Development and Characterization of Novel In-Situ-Forming Oleogels

**DOI:** 10.3390/pharmaceutics15010254

**Published:** 2023-01-11

**Authors:** Anne Dümichen, Henrike Lucas, Marie-Luise Trutschel, Karsten Mäder

**Affiliations:** Institute of Pharmacy, Martin Luther University Halle-Wittenberg, 06120 Halle (Saale), Germany

**Keywords:** in-situ-forming implant, oleogel, in-situ-forming, gel, texture analysis, rheology, syringeability, cytotoxicity, ISFO, ISFI

## Abstract

PLGA-based in situ forming implants (ISFI) often require a high amount of potentially toxic solvents such as N methyl-Pyrrolidone (NMP). The aim of the present study was to develop lipid in-situ-forming oleogels (ISFOs) as alternative delivery systems. 12-Hydroxystearic acid (12-HSA) was selected as the oleogelling agent and three different oleoformulations were investigated: (a) 12-HSA, peanut oil (PO), NMP; (b) 12-HSA, medium-chain triglycerides (MCT), ethanol; (c) 12-HSA, isopropyl myristate (IPM), ethanol. The effects of the 12-HSA concentration, preparation method, and composition on the mechanical stability were examined using a texture analysis and oscillating rheology. The texture analysis was used to obtain information on the compression strength. The amplitude sweeps were analyzed to provide information on the gel strength and the risk of brittle fractures. The frequency sweeps allowed insights into the long-term stability and risk of syneresis. The syringeability of the ISFOs was tested, along with their acute and long-term cytotoxicity in vitro. The developed ISFOs have the following advantages: (1) the avoidance of highly acidic degradation products; (2) low amounts of organic solvents required; (3) low toxicity; (4) low injection forces, even with small needle sizes. Therefore, ISFOs are promising alternatives to the existing polymer/NMP-based ISFIs.

## 1. Introduction

The development of parenteral controlled release drug delivery systems (CR-DDS) is a very attractive and rational approach to overcome several problems related to pharmacotherapy, e.g., poor or highly variable drug absorption after oral administration and short drug half-lives [1]. In addition, CR-DDS are also useful to overcome the problems of poor patient compliance [2,3]. In industrial countries, only half of all chronically ill patients adhere to their treatment plans, and the percentage is even lower in emerging and developing countries [4].

The main dosage forms of CR-DDS include preformed implants [5,6], microparticles [7,8], and in-situ-forming implants (ISFIs) [9,10,11]. Preformed implants are made via melt extrusion, which is a continuous and well-established process in the pharmaceutical industry. However, this application requires the use of large needles, which can cause anxiety and pain in patients. Microparticles, compared to implants, are more complex in their production. They can be injected with smaller cannulas, but are difficult to remove after injection. ISFIs combine the advantages of being minimally invasive and site-specific with the possibility for their removal in an emergency. ISFIs can be classified into three groups according to their implant-forming mechanism: (a) crosslinking systems; (b) phase-separating systems; (c) solidifying organogels [12]. The challenges of ISFIs include the control of the implant shape and the toxicity of the additives required for the ISFI (e.g., the toxicity of the cross-linking agents or groups and organic solvents).

The oldest ISFI system on the market is Atrigel^®^. It is a poly(lactic-co-glycolic) acid (PLGA)-based solution that is mixed with the drug in a two-syringe system prior to use. One syringe contains the solid drug and the other one contains the delivery system. Using NMP as the solvent to reduce the viscosity, the formulation can be applied as a liquid. After application into the subcutaneous (s.c.) tissue, the NMP diffuses into the surrounding tissue and water mixes with the NMP. The solvent exchange causes precipitation of the PLGA polymer and the drug. PLGA and PLA polymers are the most commonly used biodegradable materials for CR-DDS. However, the disadvantages of these polymers include their degradation to highly acidic monomers, which might trigger autocatalytic processes [13,14,15], drug degradation [16], or drug precipitation [17] prior to release due to the formation of highly acidic microenvironments [13] inside the polymer matrix. Solid lipids [18,19], organogels [20,21,22], and phospholipids [23,24] are biodegradable and biocompatible alternatives to PLGA and PLA, which deserve further investigation.

Although carcinogenic, teratogenic, and even mutagenic effects have been reported [25,26,27,28], NMP is commonly used a solubilizer and cosolvent due to its high solubilizing capacity. Even though the results of toxicity studies on organic solvents are often conflicting, in all cases lower amounts of solvents correlate with lower toxicity [29]. Commercially available Atrigel^®^-ISFIs contain quite high NMP levels ranging from 44% to 64% [30,31,32].

The aim of this study is to explore the possibilities for lipid based ISFOs as alternatives for PLGA/NMP systems. The new systems should require either lower amounts of NMP or alternative solvents. ISFOs, like conventional organogels, consist of a gelling agent and a liquid lipid matrix that form a bi-coherent system. To prevent gelling prior to injection, a solvent is added to keep the gelling agent in solution. We selected 12-HSA as the gelling agent, because it did show good oleogelling properties in food lipids [33] and it is derived from a natural source (12-HSA is obtained via the hydrogenation of ricinoleic acid, the main fatty acid in castor oil). Although 12-HSA is not yet approved for parenteral use, Kolliphor ELP, a glycerol polyethylene copolymer with ricinoleate side chains, and Kolliphor HS 15, a block polymer of polyglycol with mono- and diesters of 12-HSA, are used as excipients in parenteral formulations approved by the U.S. Food and Drug Administration (FDA) [34,35]. Therefore, parenteral use also appears feasible for 12-HSA. In addition, the results of our previous studies did show the good performance of 12-HSA-based oleogels to achieve controlled release in vitro and in vivo [20,36,37].

PO and NMP were chosen as one oil matrix and solvent combination, following previous publications [36], but lowering the required solvent content. Ethanol was tested as an alternative solvent. Since PO is only miscible with ethanol at temperatures higher than 70 °C [38], MCT and IPM were chosen as alternative liquid lipids. In order to optimize the preparation method, two different preparation methods were compared: the 12-HSA was either (a) cryomilled or (b) melted, before being mixed with the solvent and liquid lipid. Solvent-free organogel counterparts were also prepared as solvent-free references. The organogels contain neither NMP nor ethanol, but they are too viscous to be injected because the gel is already formed. In an ideal scenario, the oleogels formed from the ISFOs after exposure to the buffer would have the same gel properties as the preformed oleogels. However, the resulting gel strength depends not only on the sample composition but also on the spatial arrangements of the gel-forming agent, which can depend on the gel-forming process and the process parameters.

The solidified gels were mechanically stressed using a texture analysis and oscillating rheometry. To maximize the conclusions drawn from the rheology data, an approach following the standard of the German Institute for Standardization Registered Association (DIN) DIN 1810-2 was chosen. It provides insights into the risk of brittle fractures, syneresis, and the relative long-term stability. Based on these measurements, a manufacturing method was selected. The remaining formulations were evaluated for their acute and long-term cell toxicity and syringeability.

## 2. Materials and Methods

### 2.1. Materials

The 12-HSA (12-hydroxyoctadecanoic acid) was kindly donated by Alberdingk Boley GmbH, Krefeld Germany (12-HSA Flakes 81); the MCT by IOI Oleo GmbH, Hamburg Germany (Miglyol^®^ 812 N); and the IPM (Pionier IPM) by Hansen and Rosenthal GmbH and Co. KG, Hamburg Germany. The PO (Oleum Arachidis raffinat.) was purchased from Caesar and Loretz GmbH, Hilden Germany; the NMP (1-Methyl-2-pyrrolidinone) from Sigma-Aldrich Chemie GmbH, Taufkirchen Germany; and the ethanol from Brüggemann Alcohol Heilbronn GmbH, Heilbronn Germany. The phosphate-buffered saline pH 7.4 (PBS pH 7.4) was prepared using NaCl purchased from ORG Laborchemie GmbH, Germany; KCl from Grüssing GmbH, Filsum Germany; and KH_2_PO_4_ and Na_2_HPO_4_ × 2H_2_O from Carl Roth GmbH + Co. KG, Karlsruhe Germany. As a dialysis membrane, regenerated cellulose with a molecular weight cutoff of 10−20 kDa purchased from Carl Roth GmbH + Co. KG, Karlsruhe Germany (Nadir^®^) was used. The components for the cell toxicity studies, namely Dulbecco’s modified Eagle’s medium high-glucose (DMEM with 4500 mg/L of glucose, L-glutamine, with or without sodium pyruvate, and sodium bicarbonate), fetal calf serum (FCS), penicillin–streptomycin (with 10,000 units of penicillin and 10 mg of streptomycin per mL in 0.9% NaCl), trypsin–EDTA, and Dulbecco’s phosphate-buffered saline, as well as the fluorescent dye resazurin sodium salt and the Triton^TM^ X-100, were purchased from Sigma-Aldrich Chemie GmbH, Taufkirchen Germany.

### 2.2. Pretests

All samples contained 12-HSA at concentrations of 5, 7, 10, or 15% *m*/*m*. The concentrations refer to the final concentrations in the solidified gel without the solvent. In the following sections, “mixing” refers to blending by vortexing (MS 3 basic, IKA, Staufen Germany) at 3000 rpm. The required amount of 12-HSA was determined based on the critical gelation concentration. Increasing amounts of 12-HSA (10–250 mg in 10 mg increments) were weighed and mixed with 1 g of the respective liquid lipid matrix. The flow behavior was determined using the inversion method [39]. The critical gelling concentration is the lowest concentration of the gelling agent required that prevents the flow when turning the sample upside down. The solubility of 12-HSA in the respective solvents was assessed similarly. Increasing amounts of 12-HSA were mixed with 1 g of the respective solvent and then stored in a climate chamber at 25 °C. Polytetrafluoroethylene septa were added to the lids to prevent evaporation. After two weeks, the samples were visually inspected for the gelation and recrystallization of the 12-HSA. The solubility was determined to be the lowest concentration that did not exhibit any of these visual signs.

### 2.3. Sample Preparation

Based on the results of the pretests, the compositions shown in Table 1 were selected. The formulations are referred to by their final 12-HSA concentration in the formed gel after solvent release, since only the final concentration is important for the mechanical stability. Table 1 shows the mass fractions in the initial liquid ISFO formulations. The solvent-free oleogels have the same ratio of 12-HSA to liquid lipid, but without the solvent.

### 2.4. Preparation Methods

Each formulation was prepared in three different ways: (a) as a conventional organogel as a solvent-free reference; (b) as a cryomilled ISFO; (c) as a melted ISFO. The preparation methods are explained in more detail below.

In the following work, the samples are referred to as the amount of 12-HSA to liquid lipid with the preparation method, e.g., 5-PO-organogel or 10-melted-IPM-ISFO. A complete overview of the nomenclature used can be found in Table A1 in the Appendix A. In this subsection, “heated” refers to heating the sample above the melting point of 12-HSA:

(a) Organogel: The 12-HSA and matrix were weighed, heated, and mixed (ethanol and NMP-free systems);
(b) Melted ISFO: The 12-HSA and the solvent were weighed, heated, mixed, and cooled to room temperature. Polytetrafluoroethylene septa were added to the lids to prevent evaporation. The liquid lipid matrix was added and the entire formulation was mixed;
(c) Cryomilled ISFO: The 12-HSA flakes were cryomilled using a cryogenic mill (Cryomill, Retsch, Haan Germany). After precooling for 3 min at 1 Hz, the 12-HSA was cryomilled in three cycles for 1.5 min each at 30 Hz with intermediate pauses for 1 min each at 5 Hz. The cryomilled 12-HSA and solvent were weighed and mixed, the liquid lipid was added, and the final formulation was mixed again.

### 2.5. Preparation of the Wafers

**Figure A1:**
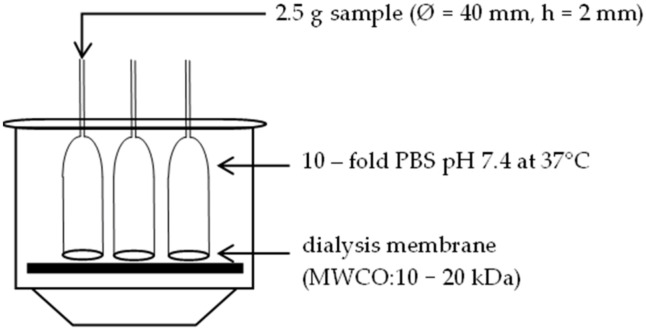
The setup for the gel wafer preparation. Upside down glass funnels were covered with dialysis membranes, filled with 2.5 g of sample, and solidified in a desiccator used as a water bath. The water bath was filled with PBS at pH 7.4 with at least 10x the volume of the gel being solidified. The buffer was heated to 37 °C. The gelling process took 48 h and the buffer was exchanged after 24 h.

An illustration of the experimental setup can be found in Figure A1 in the Appendix A. To ensure uniformity, the gels are solidified in inverted glass funnels that served as molds. The solvent release and solidification are ensured by covering the bottom of the funnels with a dialysis membrane. The funnels are placed in an empty desiccator, which serves as a water bath. The desiccator is filled with PBS at pH 7.4 to at least 10 times the volume of the samples. The buffer is heated to 37 °C and magnetically stirred to mimic the in vivo conditions. The prepared funnels are filled with 2.5 g samples from above through the thin opening of the funnel using Pasteur pipettes. The wafers solidify for 48 h and the PBS pH 7.4 is changed after 24 h.

### 2.6. Characterization of the Solid Gel Wafer

#### 2.6.1. Compression of Gel Wafers

The strength of the gel under compression was analyzed using a texture analyzer (CT3-4500, AMETEK Brookfield, Middleborough, MA, USA). The gel wafers were placed on a TA-RT-KIT fixture and compressed with a TA43 spherical probe to account for uneven surfaces in the samples. The wafers were compressed by 2.0 mm, with measurements starting at a trigger point of 0.050 N and a test speed of 2 mm/s. All samples were measured in triplicate.

#### 2.6.2. Oscillatory Rheometry

The rheology measurements were performed using an oscillating rheometer (Kinexus lab+^®^, Netzsch, Selb Germany) equipped with a cone–plate geometry (diameter: 2 cm; shearing gap: 2 mm; angle: 1°) and a heating unit. Gel slices with a diameter of 2 cm were punched out of the wafers prepared according to Section 2.5. After loading the samples, they were heated up to 37 °C to simulate the in vivo conditions. The measurements began after the temperature was constant for 10 min, which also gave the sample time to recover after loading. A strain-controlled amplitude sweep at 1 Hz was performed within a shear range of 0.001% to 1%. The frequency sweeps were executed at 0.003% shear strain and with frequencies between 0.03 and 100 Hz. Following each frequency sweep, an additional amplitude sweep was performed to ensure that the measurements were within the linear viscoelastic region (LVER). All samples were measured in triplicate.

### 2.7. Characterization of the Solution

Based on the data obtained from the solidified gels, a manufacturing method was selected and the following experiments were conducted only with samples prepared using one preparation process.

### 2.8. Syringeability

The solution was filled into a 3 mL Braun-Inject syringe and attached with different cannulas (20, 22, 24, 26 G). The syringe was then positioned underneath the texture analyzer (CT3-4500, AMETEK Brookfield, Middleborough, MA, USA) using two tripods. The TA4/1000 D cylindrical probe was adjusted to push the plunger to eject the 1 mL sample, which corresponded to a 14 mm distance. The syringe was compressed at a test speed of 0.50 mm/s measurement, which began at a trigger point of 0.005 N. All samples were measured in triplicate.

### 2.9. Cytotoxicity Studies

Two cell lines, mouse embryonic fibroblasts (3T3) and normal human dermal fibroblasts (NHDF), and two exposure periods, 24 and 96 h, were studied.

The cells were grown in their respective media, the compositions of which are shown in Table 2.

Here, 96-well plates were used as incubation vessels and filled with 100 µL of cell suspension containing 1.0 × 10^4^ 3T3 or 2.0 × 10^4^ NHDF for 24 h of exposure and 5.0 × 10^3^ cells of each cell type for 96 h of exposure. The plates were incubated at 37 °C and 5% CO_2_ for 24 h. Subsequently, 100 µL of the respective test solutions were added to each well.

**Figure A2:**
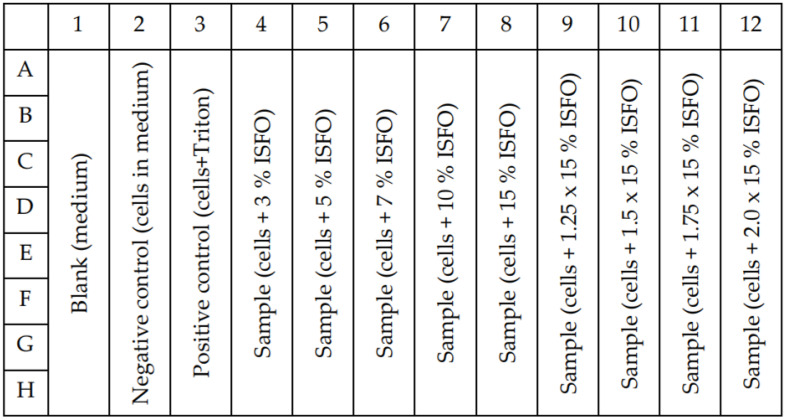
The cell toxicity testing setup. On a 96 well plate, each column is dedicated to a different test solution. Column 1 is filled with pure cell medium as a blank, column 2 contains cells in medium as a negative control, and column 3 contains cells in medium stressed by a 0.05% Triton^TM^ X 100 solution. Columns 4–8 contain cells in medium treated with test solutions corresponding to increasing 12-HSA-concentrations (3–15%). Columns 9–12 are treated with a corresponding 12-HSA-concentration to column 8 but at higher *v*/*v* ratios.

The toxicity of the ISFOs is due to the acute toxicity of the solvent and the long-term toxicity of the gel itself. Therefore, the cytotoxicity was evaluated with a concentration series of the solvent as a direct-contact test and an extraction series of the solidified gel according to the International Organization for Standardization (ISO) ISO 10993 recommendation. An illustration of the setup can be found in Figure A2 in the Appendix A. Pure medium without cells, ergo 200 µL of pure medium, was added to column 1 of the 96-well plate as a blank. Cells in medium were used as a negative control and cells stressed with 0.05% Triton^TM^ X-100 solution (*v*/*v*) were used as a positive control in columns 2 and 3, respectively. Nine columns for varying samples were available. Each sample was prepared with increasing amounts of 12-HSA, starting with 3% to 15%. The selected concentration corresponded to an injection of 200 μL of gel in 800 μL of medium, so a drug extract ratio of 0.25 was used following the ratio proposed in part 12 of ISO 10993. Since gels are defined as semisolids containing less than 15% of gelator molecules [40], gelator concentrations higher than 15% were not investigated. Instead, the last four columns were prepared using a higher volume of gel (250, 300, 350, 400 μL) injected into the same volume of medium (800 µL), or in other words the drug extract ratio was increased (0.3125; 0.375; 0.4375; 0.5).

For the solvent concentration series, the amount of solvent released from a gel was diluted within the appropriate medium and then mixed.

For the extraction series, each gel was prepared, injected into PBS at pH 7.4 and 37 °C, and shaken in a thermomixer for 24 h. The buffer was then removed and replaced with the same amount of cell medium and extracted in the thermomixer for another 24 h. Finally, the extract was isolated using a syringe with a fine needle and used as the test solution. The plates were incubated at 37 °C and 5% CO_2_ for 24 or 96 h. At the endpoint, 20 μL of 440 μM resazurin solution was added to each well and mixed. The plates were incubated for another 2 h. Resazurin is processed by viable cells into the fluorescent compound resorufin. The fluorescence intensity was measured using a multimode microplate reader (Cytation^TM^ 5 imaging reader, Agilent Technologies, Santa Clara, CA, USA) with 544 nm of excitation and 590 nm of emission. All measurements were performed in triplicate.

## 3. Results

### 3.1. Pretests

Table 3 shows the critical gelling concentration of 12-HSA in the tested liquid lipids and its solubility in the studied solvents. IPM required the highest amount of gelling agent at 4.75%, so organogels containing at least 5% 12-HSA were investigated. As mentioned above, the gels are defined as containing less than 15% of a gelling agent. Therefore, 12-HSA concentrations between 5 and 15%, more specifically 5, 7, 10, and 15%, were selected for further studies. The solubility of 12-HSA in NMP at room temperature is 400 mg g^−1^ and in ethanol is 360 mg g^−1^. Consequently, 2.5× the amount of 12-HSA was weighed in NMP and 2.8× the amount of ethanol. All solvents and lipid matrices tested were completely miscible at all ratios.

### 3.2. Characterization of the Solid Gel Wafer

#### 3.2.1. Compression of Gel Wafers

**Figure 1:**
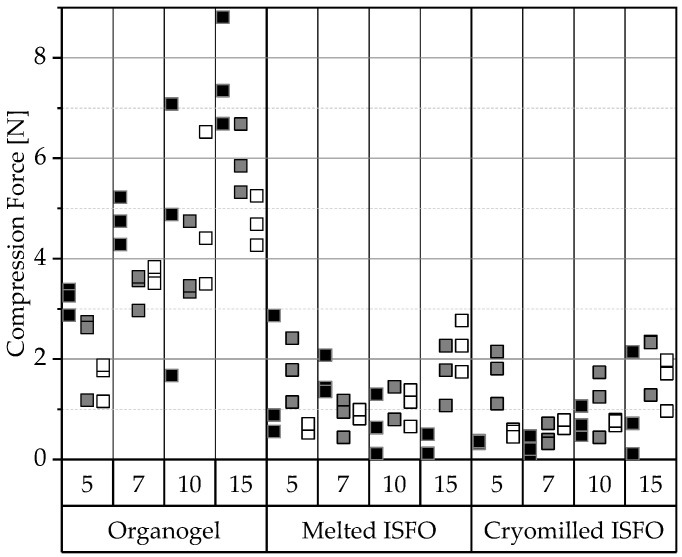
The compression force required to create a 1 mm indentation. The force required to indent the gel wafer by 1 mm was extracted from the force–displacement-curves. The data are categorized by the manufacturing process and 12-HSA concentration. The compositions of the gels are coded by the liquid lipid used (PO 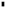, MCT 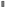, IPM 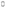).

**Figure A3:**
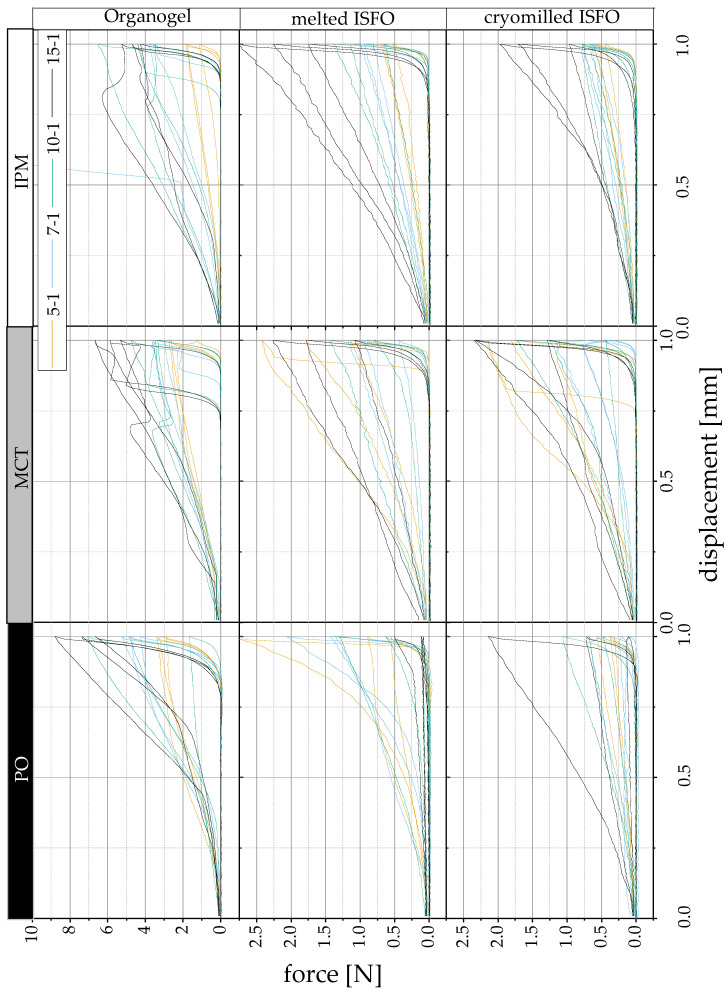
A comparison of all force–distance curves acquired via the texture analysis of the gel wafers. All force–distance curves are categorized based on their manufacturing methods in the columns and their lipid components in the rows. All triplicate measurements are shown in the same color.

Since the ISFOs are intended to be injected s.c. for prolonged release, they will be subjected to various forces over an extended period of time. One of these forces is compression, which is generated by two opposing forces that compact the sample, which can be mimicked using a texture analysis. All force–displacement curves obtained by texture analysis can be found in Figure A3 in the Appendix A. To evaluate the stability under compression, the average forces required to compress a specimen by 1 mm were compared, as shown in Figure 1.

Conventional organogels exhibit higher compressive strengths than their ISFO counterparts. An explanation can be found in the different solidification processes; the trigger for the gel formation of a 12-HSA organogel is the drop of the gel temperature below the melting point of 12-HSA, while for ISFOs the solvent has to be released. However, to form a strong gel, the hydroxyl groups of 12-HSA need time to self-assemble into a fibrillar network [41]. Cooling a preheated solution without additional methods takes longer than the almost instantaneous diffusion of the solvent. Therefore, the 12-HSA has more time to arrange in conventional organogels, resulting in higher gel strength under compression.

Higher gel strengths are expected with increasing amounts of gelling agent [42,43]. This was confirmed for organogels and the IPM samples, yet for the melted PO-ISFOs and MCT-ISFOs, the 5% samples were the strongest. This was again due to the curing process. For an ISFO to solidify, the solvent must either evaporate into the air or diffuse into a liquid, both of which are rapid processes. The available time may not be sufficient to realize the full potential of the gel strength, meaning the limiting time may outweigh the influence of the gelling agent concentration on the gel strength of ISFO.

Comparing the tested ISFOs, the impact of the manufacturing method on the gel strength is negligible. PO forms the strongest organogel network, which cannot be seen for ISFOs. IPM-ISFOs exhibit the highest reproducibility, as indicated by the nearly perfect triangular force–displacement curves found in Figure A3 of the Appendix A. MCT-containing samples show small dips in their force–displacement curves, probably because they trap more air during solidification, resulting in uneven surfaces.

Gels have been found to cause increasing inflammation at the s.c. injection site with increasing compression strength [44]. For comparison, rats tolerated chitosan gels with strengths ranging from 0.137 to 0.195 N [45] and 4% pectin gels with 0.615 N well [44]. Unfortunately, no data correlating the strength under compression and the tolerance of s.c. injectables is available. However, to avoid foreign body sensations, the compressive strength of the ISFOs should not exceed the compressive strength of the physiological tissue into which they are injected.

The adipose tissues of pork, beef, and lamb required 1.69, 4.92, and 11.6 N, respectively, to be indented by 1 mm at room temperature [46]. The ISFOs required forces of up to 3 N, which are lower than those of bovine and lamb fat tissues. The ISFOs will be presumably well tolerated, but in vivo tests are needed to confirm this hypothesis.

#### 3.2.2. Oscillatory Rheology

##### Amplitude Sweep

**Figure 2:**
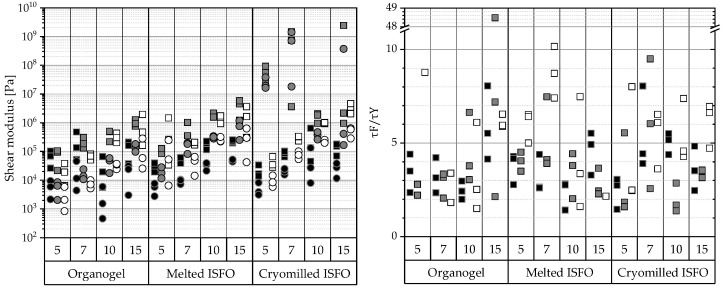
**Left**: Elastic (*G*′: 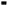) and viscous component values (*G*″: 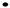). **Right**: Ratios of the flow point ($\tau_{F}$) and the yield point ($\tau_{\gamma }$ ). From amplitude sweeps acquired via oscillating rheometry, the shear moduli of *G*′ and *G*″ within the LVER and flow ($\tau_{F}$ ) and yield point ($\tau_{\gamma }$ ) were extracted. The data are categorized by the manufacturing technique and 12-HSA concentration. The compositions of the gels are coded based on the liquid lipid (PO 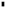, MCT 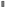, IPM 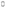).

**Figure A4:**
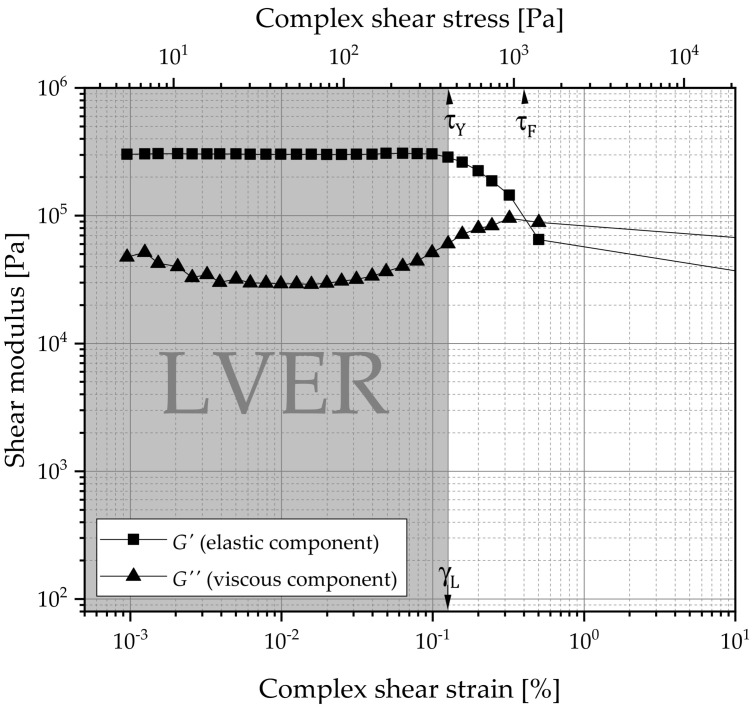
An illustration of an analysis of an amplitude sweep. As an example, the strain-controlled amplitude sweep of a 10-IPM-organogel is shown, measured at 1 Hz within a shear range of 0.001% to 1% at 37 °C. The analyzed parameters $\gamma_{L},\;\tau_{Y},\;\tau_{F}$, and LVER are marked. The end of the LVER is called the linearity limit. It is determined by *G*′, the elastic component of the sample, as it shows a plateau within the LVER. Following DIN 1810-2, the last value of *G*′, which is within a 10% range of the plateau *G*′, is the end of the LVER. The related shear strain is the linearity limit $\gamma_{L}$ and the related shear stress is called the yield stress or yield point $\tau_{Y}$. When the sample starts to flow, *G*″, the viscous component, outweighs the elastic component, which is why it is called the flow point $\tau_{F}$. By definition, this is the intersection point of *G*′ and *G*″, and the required stress for the sol–gel transition is the flow stress $\tau_{F}$.

To rheologically characterize a specimen in its natural state, a test must be performed at a stress or strain value within the linear viscoelastic region (LVER), which is the range of strain a specimen can withstand without destroying its internal structure. The amplitude sweeps can not only determine the LVER, but can also provide information about the mechanical properties and stability. This information was extracted, and an illustration and a brief explanation of the analysis can be found in Figure A4 in the Appendix A. Gel-like behavior is defined as behaving like a rheological solid, where *G*′ (elastic component) > *G*″ (viscous component) within the LVER. With the exception of the 7 and 15% cryomilled MCT samples, all exhibited this response, as shown in the left half of Figure 2.

The value of the elastic component within the LVER is also referred to as the gel strength under oscillation. All gels showed increasing strength with increasing 12-HSA concentrations, except for the PO-organogel and the cryomilled-MCT samples. In contrast to the compression tests, the ISFOs exhibited under oscillation similar or even higher gel strengths than their corresponding organogels.

Under oscillating stress at a frequency of 1 Hz porcine, the bovine and lamb adipose tissues showed *G*′ values ranging from 8.6 to 9.3 × 10^5^ Pa at room temperature [46]. Wijarnprecha et. al. measured the values at 20 °C and 80 °C, while the values in this study were determined at 37 °C. The corresponding values at 37 °C should be in between the values at 80 °C (5.53–6.78 10^5^ Pa) and 20 °C, but closer to the 20 °C values. For simplification, 10^6^ Pa was set as the upper limit for the *G*′ values within the LVER to avoid foreign body reactions. All PO samples, the 5-, 7-, and 10-MCT-organogels; and the 5-melted-MCT-ISFO complied with this demand. From the IPM samples, the 5-, 7-, and 10-organogels; the 7-melted-ISFO; and the 5- and 7-cryomilled-ISFO samples met the requirements.

The brittle fracture of an implant during its release period leads to surface and mass erosion and to the burst release of active ingredients. Depending on the loaded API, this can result in overdosing and gross overreactions. The closer the yield and the flow point are, the closer to *τ_F_*/*τ_γ_* and the more brittle the fracture is. Therefore, a high ratio is desirable. The calculated ratios of the measured specimens are shown in the right half of Figure 2. Only the IPM samples show ratios of 5 and above in all three samples of each triplicate measurement, namely the 5-IPM-organogel, 15-IPM-organogel, and 7-melted-IPM-ISFO.

##### Frequency Sweep

**Figure A5:**
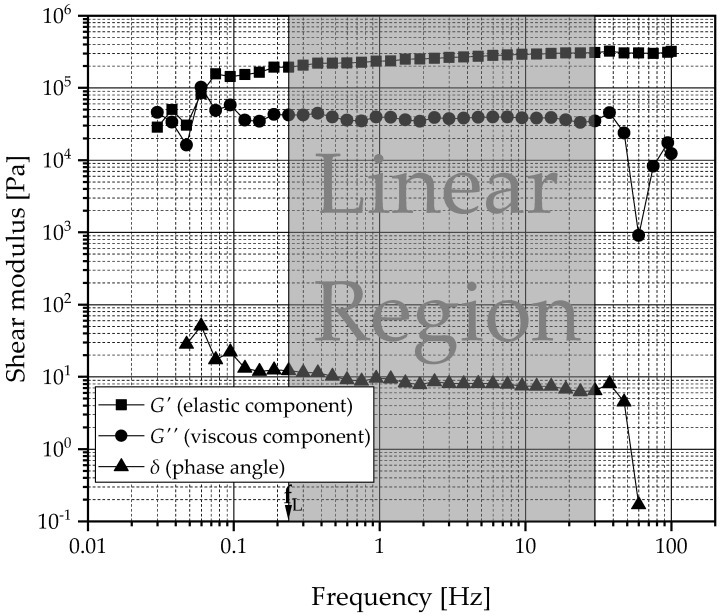
An illustration of an analysis of a frequency sweep. As an example, the frequency sweep of a melted 10-IPM-ISFO is shown, measured at a 0.003% shear strain over frequencies between 0.03 and 100 Hz at 37 °C. The analyzed parameter $f_{L}$ is marked and the linear region is shown as a gray shaded area. To compare the data, the end of the linearity was determined by fitting the *G*′-data using a least-squares linear fit. The frequency of the first data point that exceeds an error of 10% is referred to as the frequency of the linearity limit $f_{L}$. For emulsions and gels, the tendencies for syneresis can be estimated by the value of the *tanδ*; hence, the average of *δ* in the linearity range was determined and *tanδ* calculated.

Frequency sweeps are used to study time-dependent behavior. Low frequencies simulate slow, long-lasting movements and provide information about the long-term stability. For an explanation and illustration of the method used to analyze the frequency sweeps, see Figure A5 in the Appendix A.

**Figure 3:**
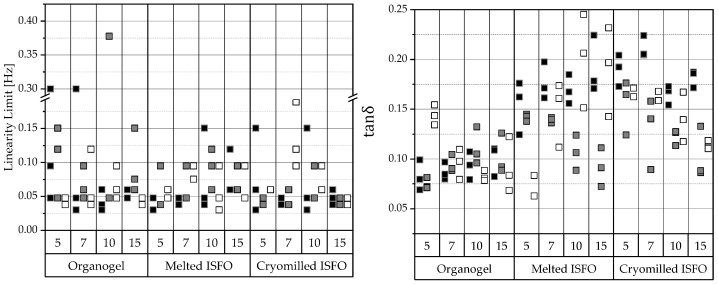
**Left**: Linearity limits. **Right**: Tangent of the phase angle (*tanδ*). The respective linearity limits and *tanδ* values within the linear range were extracted from the frequency sweeps obtained using oscillatory rheometry. The data are categorized according to the manufacturing technique and 12-HSA concentration. The compositions of the gels are coded based on the liquid lipid (PO 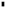, MCT 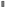, IPM 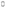).

Gels are typically independent of the frequency with respect to their phase angle. All samples show gel-like behavior, seen in 3 parallel lines of *G*′, *G*″, and *tanδ*. To compare the long-term stability of the samples, their frequencies at the linearity limit were compared, as shown in the left half of Figure 3. The lower the linearity limit, the longer the gel can be stored without any structural changes. Since most data points above 70% are between 0.03 and 0.06 Hz, which are the 4 lowest frequencies tested, there appears to be little difference between the samples in their long-term stability.

Sometimes gels tend to harden over time and their inner fluid separates—a process called syneresis. To avoid syneresis, the gel must remain flexible during storage. This can be seen in small ratios of $G^{\prime }/G^{\prime \prime }$ below 10 or if $tan\delta =G^{\prime \prime }/G^{\prime }$, which is also evident in tanδ values above 0.1, whereby the higher the value the better. The results of the analysis are shown in the right half of Figure 3. According to the results, most organogels are expected to show syneresis, but most ISFOs show tanδ>0.1 and there should be no syneresis in ISFOs, except for 5-melted-IPM-ISFO, the 10- and 15-melted-MCT-ISFO samples, and the 7- and 15-cryomilled-MCT-ISFO samples.

### 3.3. Characterization of the Solution

The following experiments were performed only with melted ISFOs based on the findings in Section 2.4. In summary, this method is easier to scale, less time-consuming, and produces better or at least similar results to cryomilling.

#### 3.3.1. Syringeability

**Figure 4:**
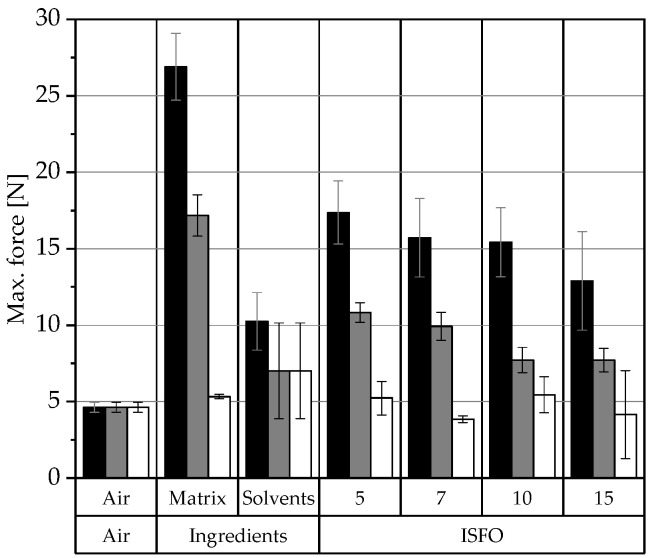
The syringeability of the different ISFOs with a 26 G cannula. The maximum force required to compress the syringe and eject the sample was extracted from the force–displacement curves obtained via the texture analysis. The data are presented as arithmetic means ± the standard deviation (SD); *n* = 3. The data are categorized by manufacturing method and 12-HSA concentration. The compositions of the gels are coded based on the liquid lipid used (PO 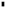, MCT 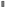, IPM 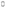). The pure liquid lipid and solvents were also ejected for comparison.

**Figure A6:**
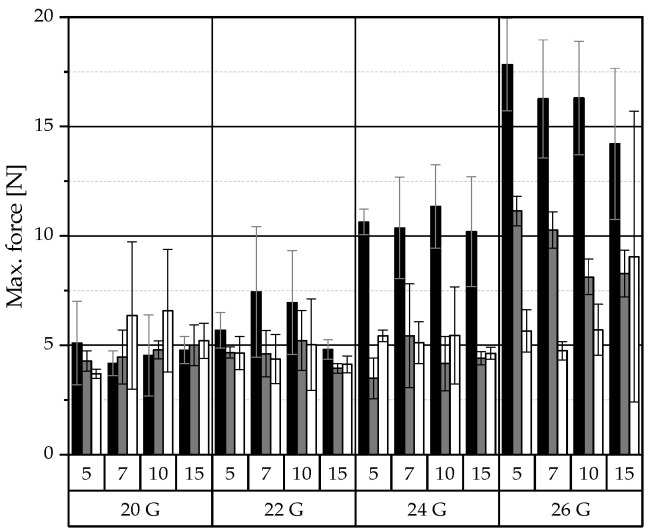
The syringeability levels using 20, 22, 24, and 26 G cannulas. The maximum forces required to compress the syringe and eject the sample were extracted from the force–distance curves acquired from the texture analysis. The data are presented as arithmetic means ± SD; *n* = 3. The data are categorized by the manufacturing technique and 12-HSA concentration. The compositions of the gels are coded based on the liquid lipids that are used (PO 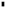, MCT 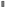, IPM 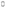).

The injectability process consists of two parts: the force required to eject the sample from the syringe (the syringeability) and the force required to inject the sample into the injection site. To minimize the used resources, the ejection forces, i.e., syringeability, were measured objectively and related to subjective injection pain information from literature. The larger the outer diameter (OD) of the cannula used, the greater the injection pain and bleeding risk [43,44]. Therefore, the smallest possible cannula should be used. All samples could be expelled with 20, 22, 24, and 26 G cannulas. The OD values decrease from 20 > 26 G; therefore, the syringeability results with the smallest 26 G cannula are the most challenging and can be seen in Figure 4. The results using the larger cannulas can be found in Figure A6 in the Appendix A.

PO samples require the highest forces for ejection, MCT the second highest, and IPM the lowest. This is also consistent with the order of viscosity and supports the hypothesis that the syringeability of ISFOs is indirectly dependent on the viscosity of the lipid matrix, since it is the main component of the formulation [47]. The force required to expel air is the force required to move the plunger, called the plunger–stopper breakloose force [48]. Surprisingly, the 7-IPM-ISFO requires even less force than air, which is likely due to the reduced friction caused by the lubrication of the plunger–syringe impact surface. The addition of solvents to the liquid lipid reduces the viscosity of the PO and MCT and the maximum force required. Since the required force for IPM is already low, this effect was not observed for all samples.

According to the in vitro and in vivo correlations found by Robinson et al. [49], all MCT- and IPM-ISFO samples can be injected easily (<12 N), while the PO samples can only be injected with considerable effort (<38 N). To minimize the injection pain, the maximum injection force should be less than 20 N [50], which was the case for all samples. Formulations requiring less than 10 N can even be injected with smaller cannulas [51], so the IPM samples and the 10- and 15-MCT samples can even be injected with a 27 or 28 G cannula.

Currently, three parenteral ISFIs based on the Atrigel^®^ system, Eligard^®^, Perseris^®^, and Sublocade^®^, are FDA-approved. Cannula thicknesses of 18–20 G are intended for their use [30,31,32], corresponding to an OD range of 0.91–1.27 mm. Preformed implants, such as Implanon^®^ and Zoladex^®^, require even larger cannulas of 14–16 G with an OD range of 1.63–1.83 mm. Injecting ISFOs with a 26 G cannula (OD = 0.404 mm) reduces the OD of the cannula to one-quarter of the size used for preformed implants and one-third of the size used for polymer-based ISFIs. For the abovementioned MCT and IPM samples, which can be injected with a 27 G needle (OD = 0.361 mm), the OD can even be reduced by a factor of 5 compared to preformed implants and by a factor of 3.5 compared to the commercially available ISFIs. This drastically reduces the pain and risk of injury during injection and increases patient adherence.

#### 3.3.2. Cytotoxicity Studies

**Figure 5:**
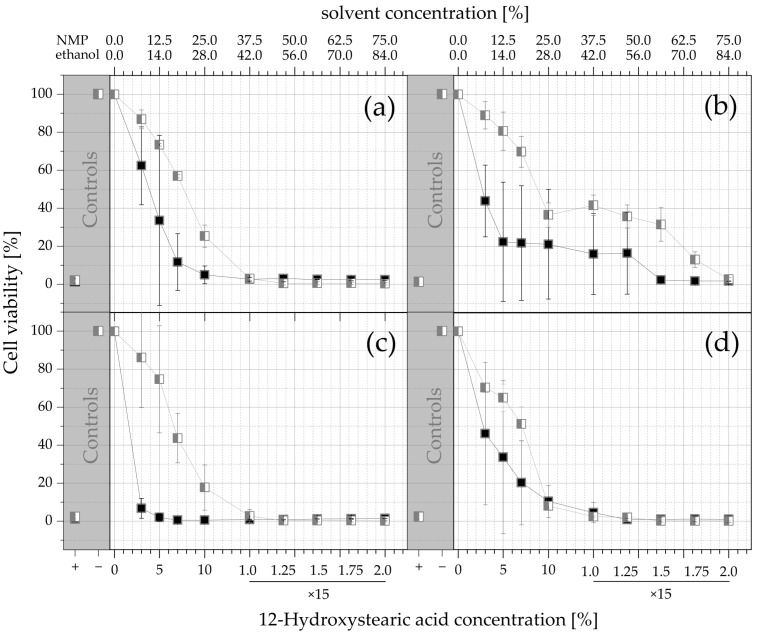
Acute toxicity–cell viability levels after exposure to solvents: (**a**) 3T3 cells after 24 h; (**b**) NHDF cells after 24 h; (**c**) 3T3 cells after 96 h; (**d**) NHDF cells after 96 h. Positive and negative controls for each assay are provided in the gray sections to demonstrate the performance of the experimental design. The cells were treated with solutions of NMP (PO ISFOs) 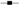 and ethanol (MCT and IPM ISFOs) 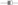. The viability values are given as a function of the solvent concentration and the corresponding 12-HSA concentration, and as arithmetic mean ± SD; *n* = 3. Since no higher 12-HSA concentrations than 15% were tested, the amount of ISFO injected into the extraction medium was increased for the last 4 datapoints (250, 300, 350, and 400 µL into 800 µL of medium; for all other datapoints 200 µL was injected into 800 µL).

The fluorescence intensities of the treated wells were background-corrected by subtracting the intensities of the blanks. The results were set in relation to the negative control (cells in medium), which was set to 100% cell viability. Linear interpolation was used to determine the half-maximal inhibitory concentration (*IC*_50_). The effects of the NMP and ethanol on the viability of 3T3 and NHDF cells after 24 and 96 h of incubation and the acute toxicity levels of the PO ISFOs and MCT/IPM ISFOs are shown in Figure 5.

Due to the constant ratio of both compounds in the formulation, the mass of 12-HSA is proportional to the mass of NMP by a factor of 2.5 and to the mass of ethanol by a factor 2.8. NMP was much more toxic than ethanol in both cases, and 3T3 cells were more sensitive than NHDF cells, as supported by the *IC_50_* values in Table 4.

For NMP and the PO-ISFOs, the *IC*_50_ values show that even the lowest concentration of NMP in the 3-PO-ISFO kills more than half of the 3T3 cells after 24 h and 96 h and the NHDF cells after 96 h of exposure. NMP is also used as the solvent in the commercial Atrigel^®^ system. For NHDF cells, the viability after exposure to the solvent of the Atrigel^®^ system is less than 20% and for 3T3 cells is even less than 5%.

In contrast to NMP, the cells can withstand ethanol amounts up to 7-MCT-ISFO and 7-IPM-ISFO with less than 50% cell death. Therefore, ethanol is much more tolerable than NMP and is a better choice as a solvent. In the short term, all ISFO systems are less toxic compared to PLGA/NMP-based systems.

**Figure 6:**
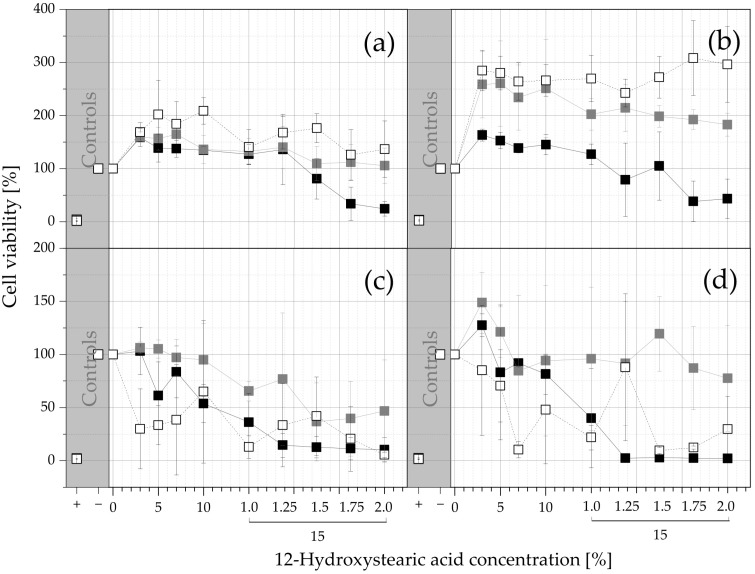
Long-term toxicity–cell viability levels after exposure to ISFO extracts: (**a**) 3T3 cells after 24 h; (**b**) NHDF cells after 24 h; (**c**) 3T3 cells after 96 h of exposure; (**d**) NHDF cells after 96 h. Positive and negative controls for each assay are provided in the gray sections to demonstrate the performance of the experimental design. The cells were treated with PO ISFO extracts (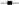), MCT-ISFO extracts (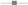), and IPM ISFO extracts (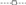). The viability values are given as a function of the 12-HSA concentrations as arithmetic means ± SD; *n* = 3. Since no higher 12-HSA concentrations than 15% were tested, the amount of ISFO injected into the extraction medium was increased for the last 4 datapoints (250, 300, 350, and 400 µL into 800 µL of medium; for all other datapoints 200 µL was injected into 800 µL).

The long-term effects of PO, MCT, and IPM extracts on the viability of 3T3 and NHDF cells after 24 and 96 h of incubation are shown in Figure 6. The toxicity is shown as a function of the 12-HSA concentration in the extracted gels. After 24 h of incubation, both treated cell lines not only survived but even thrived and outperformed the negative control in terms of the fluorescence intensity and cell viability, with values as high as 300%. In resazurin assays, the fluorescence intensity is proportional to the number of metabolically active cells over a wide concentration range [52]. This means that the cell number in the treated wells is higher than in the negative control because the treated cells proliferate.

One explanation is that the lipid components were extracted from the ISFOs and used as an additional lipid source for energy, carbon, and as building blocks, providing nourishment for proliferation, in addition to the nutrients needed for growth in the medium.

Both the 3T3 and NHDF cells show that the cell viability was increased in the order of IPM > MCT > PO extracts, with higher amplitudes in NHDF cells. Most cells require fatty acids for energy production, which is why triglycerides must be hydrolyzed before they can be used [53].

IPM is an ester of isopropyl alcohol and myristic acid; it might be more easily hydrolyzed than the triglycerides PO and MCT, thereby providing energy more rapidly and resulting in it having the highest cell viability. MCT have been shown to be hydrolyzed faster than long-chain triglycerides such as PO [54] and can provide energy faster, explaining the higher viability of cells treated with MCT extracts. After 24 h of incubation, only the PO extracts reduced the cell viability to below 50%, meaning the *IC_50_* values for the MCT and IPM samples could not be calculated and can only be stated to be above the highest tested concentration of 15% 12-HSA, with a drug extract ratio of 0.5. All calculated values can be found in Table 5.

In contrast to 24 h of incubation, where the cell viability follow the order of IPM > MCT > PO, the order is MCT > PO > IPM after 96 h of incubation. The IPM-treated cells appear to exhibit a discontinuous downward trend with increasing 12-HSA concentrations, suggesting that factors other than the toxicity of the extract may play a role. Since the cell viability of the IPM-treated cells after 24 h indicates that the cells proliferate and have a high cell density, the cell density is expected to be even higher after 96 h. Zheng et al. found that mouse fibroblasts (L929 cells) begin to reduce the fluorescent resorufin to the non-fluorescent dihydroresorufin when the cell density becomes too high [55], which would explain the low fluorescence intensity of the IPM-treated cells. High cell densities can also lead to shortages of space, nutrients, and air, resulting in cell death, and as the cell density increases, the range of optimal incubation times also becomes smaller [56].

The results after 96 h show higher SD values compared to the measurements after 24 h, which could be due to the presence of lipid droplets on the surfaces of the wells. The lipid droplets may distort the fluorescence intensity measurements, leading to unreliable and highly variable results, which would explain the high SD values for the 96 h values.

Therefore, the 24 h extract values are more reliable than the 96 h values and should be given more consideration. In summary, the acute toxicity of the PO extracts (NMP) is higher than for the MCT and IPM extracts (ethanol). The 24 h extract values indicate that PO-ISFO extracts up to 15% PO-ISFO with a drug extract ratio of 0.4375 and all tested concentrations of MCT- and IPM-ISFOs have no 96 h toxicity.

## 4. Discussion

The goal of the experiments with the solid gel wafers was to find the best manufacturing method. In order to provide an overview, a scoring system was introduced, the evaluation criteria of which are shown in Table 6.

The results for the solidified gel wafers were divided into 4 categories, which were evaluated with points from 0 to 3. Since the differences in compressive strength were negligible and all specimens had values below the compressive strength of physiological tissue, the reproducibility in terms of the SD was used for the evaluation. Since there was little difference in the long-term stability levels of the samples, linearity limits were not included in the evaluation. Each value of a triplicate measurement was evaluated, and the the lowest value was considered. In the end, the average over all measurements was calculated and the results were color-coded for better visualization, as seen in Table 7, where the more intense the color, the better.

Melted and cryomilled ISFOs show similar behaviors. Because preparation via melting is less time- and resource-intensive, this method was chosen for the ISFO preparation process. All MCT- and IPM-ISFOs can be easily injected with a 26 G cannula (<12 N). PO samples require larger forces (<38 N). All IPM samples and the 10- and 15-MCT samples can even be injected with a 27 or 28 G cannula. This reduces the OD of the cannulas to one-fifth the size of the cannulas used for preformed implants and to nearly one-fourth the size of the cannulas used for polymer-based Atrigel^®^ ISFIs. This would result in higher patient adherence.

The developed ISFO systems (particularly the ethanol-based ISFOs) are less toxic than PLGA-NMP systems in vitro. Combining the mechanical stability, syringeability, and IC_50_ values after exposure, the melted 7% IPM-ISFO is the most promising ISFO. Therefore, the 7-melted-IPM-ISFO will be investigated in further in vitro and in vivo experiments to fully understand the release kinetics, compatibility, and degradability.

In summary, 12-HSA-based in-situ-forming oleogels (ISFOs) are promising alternatives to PLGA/NMP ISFI systems. The advantages can be summarized by the following points:

- Avoidance of highly acidic degradation products;
- Lower amounts of organic solvents required;
- Low toxicity;
- Low injection forces involved even with small needle sizes (28 G).

The current study shows the high potential of the ISFOs for parenteral controlled release. The existing data show that lipase can accelerate the erosion of ISFOs in vitro [36]. The in vivo data demonstrate the erosion of ISFOs (measured via 3D ultrasounds) [36] and the local retainment of nanosized oxygen sensors over several weeks [37]. However, more in vitro and in vivo studies are needed to investigate the drug release kinetics and mechanisms and also the in vivo behavior in a broader setup and in more detail.

## Figures and Tables

**Table 1:** The compositions of the formulations selected from the pretests for further studies.

m%	PO				MCT				IPM			
nomenclature	5	7	10	15	5	7	10	15	5	7	10	15
12-HSA	4.5	6.0	8.1	11.2	4.4	5.9	7.8	10.6	4.4	5.9	7.8	10.6
liquid lipid	85.2	80.1	73.2	63.2	83.3	77.8	70.3	59.9	83.3	77.8	70.3	59.9
solvent	10.3	13.9	18.7	25.7	12.3	16.4	21.9	29.6	12.3	16.4	21.9	29.6

**Table 2:** The compositions of the cell media.

Cell Line	3T3 V [mL]	NHDF V [mL]
DMEM with Na-Pyruvate	500	-
DMEM without Na-Pyruvate	-	500
FCS (f.c. 10%)	55	55
Penicillin–streptomycin solution (100×)	5.5	5.5

**Table 3:** The critical gelling concentrations of 12-HSA in PO, MCT, and IPM and the solubility of 12-HSA in NMP and ethanol as the mass fraction composition.

m%	PO	MCT	IPM
Critical gelling concentration	2.91	3.85	4.75
**m%**	**NMP**	**ethanol**	
Solubility of 12-HSA	28.57	26.47	

**Table 4:** The half-maximal inhibitory concentration (*IC*_50_) values of the solvents themselves and expressed as the corresponding 12-HSA concentrations [%], calculated as arithmetic means ± SDs of each triplicate measurement.

Formulation	PO (NMP)		MCT/IPM (ethanol)	
Cell Type	3T3	NHDF	3T3	NHDF
**24 h incubation**				
Solvent conc. [%]	9.65 ± 3.15	6.675 ± 0.625	21.42 ± 0.728	24.584 ± 1.792
12-HSA-conc. [%]	3.86 ± 1.26	2.67 ± 0.25	7.65 ± 0.26	8.78 ± 0.64
**96 h incubation**				
Solvent-conc. [%]	4.025 ± 0.1	6.95 ± 1.3	18.452 ± 3.808	20.104 ± 1.624
12-HSA-conc. [%]	1.61 ± 0.04	2.78 ± 0.52	6.59 ± 1.36	7.18 ± 0.58

**Table 5:** The half-maximal inhibitory concentration (*IC_50_*) values of ISFO extracts expressed as the corresponding 12-HSA concentrations [%], calculated as arithmetic means ± SD of each triplicate measurement.

	PO		MCT		IPM	
Cell Type	3T3	NHDF	3T3	NHDF	3T3	NHDF
24 h	24.95 ± 1.30	25.59 ± 0.65	>30.00	>30.00	>30.00	>30.00
96 h	19.94 ± 2.56	13.01 ± 1.99	21.25 ± 1.25	>30.00	2.14 ± 0.40	5.68 ± 1.18

**Table 6:** An overview of the color-coded assessment system (parameters used in parentheses). The more points and the more intense the color, the better are the gel characteristics.

	0 Points	1 Point	2 Points	3 Points
Assessment	Very poor	Poor	Moderate	Good
**Gel strength under compression** (relative standard deviation *)	>50%	<50%	<30%	<10%
**Gel strength under oscillation** (LVER-*G*′ of amplitude sweep)	>10^8^ Pa	9.3 × 10^5^– 10^8^ Pa	<10^4^ Pa	10^4^– 9.3 × 10^5^ Pa
**Risk of brittle fracture** (*τ_F_*/*τ_γ_* of amplitude sweep)	1–2.5	2.5–5	5–10	>10
**Tendency to syneresis** (tanδ of frequency sweep)	0–0.1	0.1–0.15	0.15–0.2	0.2–0.3

* Since the differences in gel strength under compression (as the force required to create a 1 mm indentation in gel wafers) were only minimal, the reproducibility in the form of the SD was evaluated.

**Table 7:** Color-coded evaluation of gel wafer parameters (An explanation of the meaning of each color can be found in Table 6).

		Gel Strength under Compression			Gel Strength under Oscillation			Risk of Brittle Fracture			Tendency to Syneresis			Mean over All Categories		
		PO	MCT	IPM	PO	MCT	IPM	PO	MCT	IPM	PO	MCT	IPM	PO	MCT	IPM
Melted	5	0	1	2	3	3	1	1	1	2	1	1	0	1.25	1.5	1.25
	7	2	1	3	3	1	3	1	1	2	2	1	1	2	1	2.25
	10	0	1	1	3	1	1	0	0	0	2	0	2	1.25	0.5	1
	15	0	1	2	3	1	1	1	0	0	2	0	1	1.5	0.5	1
Cryomilled	5	2	1	2	3	0	3	0	0	0	2	1	2	1.75	0.5	1.75
	7	0	1	2	3	0	3	1	1	1	3	0	2	1.75	0.5	2
	10	1	0	3	3	1	1	1	0	1	2	1	1	1.75	0.5	1.5
	15	0	1	1	3	0	1	0	1	1	2	0	1	1.25	0.5	1

## Data Availability

Not applicable.

## Appendix A

**Table A1:** The nomenclature of the used formulations.

	Initial 12-HSA-conc. [%] ^1^	Final 12-HSA-conc. [%] ^2^	Liquid Lipid	Solvent	Preparation Method
5-PO-organogel	5	5	PO	NMP	Organogel
7-PO-organogel	7	7	PO	NMP	Organogel
10-PO-organogel	10	10	PO	NMP	Organogel
15-PO-organogel	15	15	PO	NMP	Organogel
melted 5-PO-ISFO	4.5	5	PO	NMP	melted ISFO
melted 7-PO-ISFO	6.0	7	PO	NMP	melted ISFO
melted 10-PO-ISFO	8.1	10	PO	NMP	melted ISFO
melted 15-PO-ISFO	11.2	15	PO	NMP	melted ISFO
cryomilled 5-PO-ISFO	4.5	5	PO	NMP	cryomilled ISFO
cryomilled 7-PO-ISFO	6.0	7	PO	NMP	cryomilled ISFO
cryomilled 10-PO-ISFO	8.1	10	PO	NMP	cryomilled ISFO
cryomilled 15-PO-ISFO	11.2	15	PO	NMP	cryomilled ISFO
5-MCT-organogel	5	5	MCT	ethanol	Organogel
7-MCT-organogel	7	7	MCT	ethanol	Organogel
10-MCT-organogel	10	10	MCT	ethanol	Organogel
15-MCT-organogel	15	15	MCT	ethanol	Organogel
melted 5-MCT-ISFO	4.4	5	MCT	ethanol	melted ISFO
melted 7-MCT-ISFO	5.9	7	MCT	ethanol	melted ISFO
melted 10-MCT-ISFO	7.8	10	MCT	ethanol	melted ISFO
melted 15-MCT-ISFO	10.6	15	MCT	ethanol	melted ISFO
cryomilled 5-MCT-ISFO	4.4	5	MCT	ethanol	cryomilled ISFO
cryomilled 7-MCT-ISFO	5.9	7	MCT	ethanol	cryomilled ISFO
cryomilled 10-MCT-ISFO	7.8	10	MCT	ethanol	cryomilled ISFO
cryomilled 15-MCT-ISFO	10.6	15	MCT	ethanol	cryomilled ISFO
5-IPM-organogel	5	5	IPM	ethanol	Organogel
7-IPM-organogel	7	7	IPM	ethanol	Organogel
10-IPM-organogel	10	10	IPM	ethanol	Organogel
15-IPM-organogel	15	15	IPM	ethanol	Organogel
melted 5-IPM-ISFO	4.4	5	IPM	ethanol	melted ISFO
melted 7-IPM-ISFO	5.9	7	IPM	ethanol	melted ISFO
melted 10-IPM-ISFO	7.8	10	IPM	ethanol	melted ISFO
melted 15-IPM-ISFO	10.6	15	IPM	ethanol	melted ISFO
cryomilled 5-IPM-ISFO	4.4	5	IPM	ethanol	cryomilled ISFO
cryomilled 7-IPM-ISFO	5.9	7	IPM	ethanol	cryomilled ISFO
cryomilled 10-IPM-ISFO	7.8	10	IPM	ethanol	cryomilled ISFO
cryomilled 15-IPM-ISFO	10.6	15	IPM	ethanol	cryomilled ISFO

^1^: The concentration of 12-HSA in the liquid solution prior to curing of the gel. ^2^: The concentration of 12-HSA in the cured gel for ISFO after solvent release.

## References

[1] Chaudhary K., Patel M.M., Mehta P.J. (2019). Long-acting injectables: Current perspectives and future promise. Crit. Rev. Ther. Drug Carrier Syst..

[2] Ringe J.D., Farahmand P. (2014). Improved real-life adherence of 6-monthly denosumab injections due to positive feedback based on rapid 6-month BMD increase and good safety profile. Rheumatol. Int..

[3] Poulos C., Kinter E., Yang J.C., Bridges J.F.P., Posner J., Reder A.T. (2016). Patient Preferences for Injectable Treatments for Multiple Sclerosis in the United States: A Discrete-Choice Experiment. Patient.

[4] Sabaté E., World Health Organization (2003). Adherence to Long-Term Therapies: Evidence for Action.

[5] Esfahani G., Häusler O., Mäder K. (2022). Controlled release starch-lipid implant for the therapy of severe malaria. Int. J. Pharm..

[6] Althobaiti A.A., Ashour E.A., Almutairi M., Almotairy A., Al Yahya M., Repka M.A. (2022). Formulation Development of Curcumin-piperine solid dispersion via hot-melt extrusion. J. Drug Deliv. Sci. Technol..

[7] Steiner J., Alaneed R., Kressler J., Mäder K. (2020). Fatty acid-modified poly(glycerol adipate) microparticles for controlled drug delivery. J. Drug Deliv. Sci. Technol..

[8] Sivasankaran S., Jonnalagadda S. (2022). Levonorgestrel loaded biodegradable microparticles for injectable contraception: Preparation, characterization and modelling of drug release. Int. J. Pharm..

[9] Thakur R.R.S., McMillan H.L., Jones D.S. (2014). Solvent induced phase inversion-based in situ forming controlled release drug delivery implants. J. Control. Release.

[10] Hui J., Wong M., Pei R., Tan T., Chang J.J., Qi B., Chan Y., Zhao X., Jian J., Cheng W. (2022). Injectable Hybrid-Crosslinked Hydrogels as Fatigue-Resistant and Shape-Stable Skin Depots. Biomacromolecules.

[11] Vintiloiu A., Lafleur M., Bastiat G., Leroux J.C. (2008). In situ-forming oleogel implant for rivastigmine delivery. Pharm. Res..

[12] Kempe S., Mäder K. (2012). In situ forming implants—An attractive formulation principle for parenteral depot formulations. J. Control. Release.

[13] Mäder K., Gallez B., Liu K.J., Swartz H.M. (1996). Non-invasive in vivo characterization of release processes in biodegradable polymers by low-frequency electron paramagnetic resonance spectroscopy. Biomaterials.

[14] Fu K., Pack D.W., Klibanov A.M., Langer R. (2000). Visual Evidence of Acidic Environment Within Degrading. Pharm. Res..

[15] Zolnik B.S., Burgess D.J. (2007). Effect of acidic pH on PLGA microsphere degradation and release. J. Control. Release.

[16] Houchin M.L., Topp E.M. (2008). Chemical Degradation of Peptides and Proteins in PLGA: A Review of Reactions and Mechanisms.

[17] Zlomke C., Barth M., Mäder K. (2019). Polymer degradation induced drug precipitation in PLGA implants—Why less is sometimes more. Eur. J. Pharm. Biopharm..

[18] Sax G., Kessler B., Wolf E., Winter G. (2012). In-vivo biodegradation of extruded lipid implants in rabbits. J. Control. Release.

[19] Vollrath M., Engert J., Winter G. (2017). Long-term release and stability of pharmaceutical proteins delivered from solid lipid implants. Eur. J. Pharm. Biopharm..

[20] Mäder K., Windorf M., Kutza J. (2014). Injectable Depot Formulations for the Controlled Release of Active Agents.

[21] Esposito C.L., Kirilov P., Roullin V.G. (2018). Organogels, promising drug delivery systems: An update of state-of-the-art and recent applications. J. Control. Release.

[22] Esposito C.L., Tardif V., Sarrazin M., Kirilov P., Roullin V.G. (2020). Preparation and characterization of 12-HSA-based organogels as injectable implants for the controlled delivery of hydrophilic and lipophilic therapeutic agents. Mater. Sci. Eng. C.

[23] Rahnfeld L., Luciani P. (2020). Injectable Lipid-Based Depot Formulations: Where Do We Stand?. Pharmaceutics.

[24] Zlomke C., Albrecht J., Mäder K. (2020). Nicardipine Loaded Solid Phospholipid Extrudates for the Prevention of Cerebral Vasospasms: In Vitro Characterization. Pharmaceutics.

[25] Jouyban A., Fakhree M.A.A., Shayanfar A. (2010). Review of pharmaceutical applications of N-methyl-2-pyrrolidone. J. Pharm. Pharm. Sci..

[26] Rowe R.C., Sheskey P.J., Owen S.C. (2006). Handbook of Pharmaceutical Excipients.

[27] Meek M.E., Walker M., Beauchamp R., Canada H. (2001). Concise International Chemical Assessment Document 35: N-Methyl-2-Pyrrolidone.

[28] Winchell C.J., Hertz S.H. (2017). Summary Review: Sublocade^®^ (Buprenorphine). Application Number: 209819Orig1s000.

[29] Strickley R.G. (2004). Solubilizing Excipients in Oral and Injectable Formulations. Pharm. Res..

[30] Tolmar Pharmaceuticals, Inc. (2018). Highlights of Prescribing Information—ELIGARD®.

[31] Indivior UK Limited (2018). Highlights of Prescribing Information—PerserisTM.

[32] Indivior UK Limited (2017). Highlights of Prescribing Information—SublocadeTM.

[33] Jiang Z., Lu X., Geng S., Ma H., Liu B. (2020). Structuring of sunflower oil by stearic acid derivatives: Experimental and molecular modelling studies. Food Chem..

[34] Gelderblom H., Verweij J., Nooter K., Sparreboom A. (2001). Cremophor EL: The drawbacks and advantages of vehicle selection for drug formulation. Eur. J. Cancer.

[35] Europe P., Keyword E.Y., Print E., License S., Velagaleti R. (2011). Solutol HS15 as a Novel Excipient Solutol HS15 as a Novel Excipient. Pharm. Technol..

[36] Windorf M. (2017). 12-Hydroxystearic Acid-Based In Situ Forming Organogels: Development and Characterization.

[37] Lampp L., Rogozhnikova O.Y., Trukhin D.V., Tormyshev V.M., Bowman M.K., Devasahayam N., Krishna M.C., Mäder K., Imming P. (2019). A radical containing injectable in-situ-oleogel and emulgel for prolonged in-vivo oxygen measurements with CW EPR. Free Radic. Biol. Med..

[38] Kaparthi R., Chari K.S. (1959). Solubilities of vegetable oils in aqueous ethanol and ethanol-hexane mixtures. J. Am. Oil Chem. Soc..

[39] Jeong B., Lee D.S., Shon J., Bae Y.H., Kim S.W. (1998). Thermoreversible Gelation of Poly (Ethylene Oxide ). J. Polym. Sci. Part A Polym. Chem..

[40] Vintiloiu A., Leroux J.C. (2008). Organogels and their use in drug delivery—A review. J. Control. Release.

[41] Fameau A.L., Rogers M.A. (2020). The curious case of 12-hydroxystearic acid—The Dr. Jekyll & Mr. Hyde of molecular gelators. Curr. Opin. Colloid Interface Sci..

[42] Burkhardt M., Kinzel S., Gradzielski M. (2009). Macroscopic properties and microstructure of HSA based organogels: Sensitivity to polar additives. J. Colloid Interface Sci..

[43] Lan Y., Rogers M.A. (2015). 12-Hydroxystearic acid SAFiNs in aliphatic diols-a molecular oddity. CrystEngComm.

[44] Markov P.A., Khramova D.S., Shumikhin K.V., Nikitina I.R., Beloserov V.S., Martinson E.A., Litvinets S.G., Popov S.V. (2019). Mechanical properties of the pectin hydrogels and inflammation response to their subcutaneous implantation. J. Biomed. Mater. Res. Part A.

[45] Kashyap N., Viswanad B., Sharma G., Bhardwaj V., Ramarao P., Ravi Kumar M.N.V. (2007). Design and evaluation of biodegradable, biosensitive in situ gelling system for pulsatile delivery of insulin. Biomaterials.

[46] Wijarnprecha K., Fuhrmann P., Gregson C., Sillick M., Sonwai S., Rousseau D. (2022). Temperature-dependent properties of fat in adipose tissue from pork, beef and lamb. Part 2: Rheology and texture. Food Funct..

[47] Dexter M.B., Shott M.J. (1979). The evaluation of the force to expel oily injection vehicles from syringes. J. Pharm. Pharmacol..

[48] Cilurzo F., Selmin F., Minghetti P., Adami M., Bertoni E., Lauria S., Montanari L. (2011). Injectability Evaluation: An Open Issue. AAPS PharmSciTech.

[49] Robinson T.E., Hughes E.A.B., Bose A., Cornish E.A., Teo J.Y., Eisenstein N.M., Grover L.M., Cox S.C. (2020). Filling the Gap: A Correlation between Objective and Subjective Measures of Injectability. Adv. Healthc. Mater..

[50] Watt R.P., Khatri H., Dibble A.R.G. (2019). Injectability as a function of viscosity and dosing materials for subcutaneous administration. Int. J. Pharm..

[51] Rungseevijitprapa W., Bodmeier R. (2009). Injectability of biodegradable in situ forming microparticle systems (ISM). Eur. J. Pharm. Sci..

[52] Kuete V., Karaosmanoğlu O., Sivas H. (2017). Anticancer Activities of African Medicinal Spices and Vegetables. Medicinal Spices and Vegetables from Africa.

[53] Tada H., Takamura M., Kawashiri M.A. (2020). Genomics of hypertriglyceridemia. Adv. Clin. Chem..

[54] Zawistowski J., Kopeć A. (2022). Effect of functional food ingredients on nutrient absorption and digestion. Nutrition and Functional Foods in Boosting Digestion, Metabolism and Immune Health.

[55] Zheng F., Wang S., Hou W., Xiao Y., Liu P., Shi X., Shen M. (2019). Comparative study of resazurin reduction and MTT assays for cytocompatibility evaluation of nanofibrous materials. Anal. Methods.

[56] Prabst K., Engelhardt H., Ringgeler S., Hubner H., Ates G., Vanhaeke T., Rogiers V., Rodrigues R., Chan L.L.-Y., McCulley K.J. (2017). Cell Viability Assays.

